# Open questions in biology - a tenth anniversary series

**DOI:** 10.1186/1741-7007-11-7

**Published:** 2013-01-31

**Authors:** Miranda Robertson

**Affiliations:** 1 BMC Biology, BioMed Central, 236 Gray's Inn Road, London WC1X 8HL, UK

## 

In November of this year, it will be ten years since *BMC Biology *published its first research paper. We shall be marking this anniversary year in April with a special collection of articles reflecting some of our most notable publications of the decade, and a very special celebration at the FASEB meeting in Boston in April - more news on that soon.

But meanwhile, and throughout the year, we shall be publishing short contributions from our Editorial Board on those open questions they find most pressing, tantalizing, or just interesting, in their fields, both to reflect the particular interests of a journal with a very broadly defined scope, and to provide some very brief and lively reading.

Some members of our Editorial Board have contributed just a paragraph or two, and these contributions will be published together in small collections: our inaugural collection [1] contains a challenge from Stephen Benkovic to researchers engaged in the high-throughput proteomic identification of protein-protein interactions; a question from Julie Theriot on the motor proteins of bacteria; and a proposal from Dagmar Ringe for 21^st^-century evolutionary biology.

Others have sent slightly longer contributions, of which we publish two this month: Gillian Griffiths on nagging puzzles in immunology, all with membrane transport at their heart [2]; and Frank Uhlmann on unanswered questions in chromosome packaging [3], not the least of which is the *modus operandi *of the structural maintenance of chromosomes (SMC) molecules, whose hinged coil-coil structure and ATPase activity must be telling us something about how they function in chromosome condensation (and sister chromatid cohesion and DNA repair), though it remains tantalizingly unclear exactly what.

## What's the question?

The questions raised by Stephen Benkovic and Dagmar Ringe both arise from the immense power of the technology that can now be applied to biological systems and the depth of understanding that can be reached of the workings of macromolecules.

Genomics and proteomics may deliver answers for which it is not so easy to define the questions. A profound understanding of the molecular mechanism of an enzyme may suggest ingenious new ways of targeting drugs without offering much clue about the side effects. These are generic issues.

## What's the answer?

Frank Uhlmann and Gillian Griffiths raise questions specific to their fields, and focused on the mechanism of defined phenomena - the condensation of mitotic chromosomes, the presentation of extracellular antigens to cytotoxic T cells and their resistance to their own cytotoxic output; and the escape of cathepsins from lysosomes in an unconventional variant of apoptosis. These are not questions that have so far yielded to the force of modern biological technology - that is why, though none of them is very new, they are unanswered. Naturally we shall welcome the submission to *BMC Biology * of any significant steps toward the solution to these problems.

The same applies to Sean Munro’s thought-provoking contribution, ‘What is there left for cell biologists to do?’, which we shall publish in February.
